# Integrated Analysis of Long Noncoding RNA Expression Profiles in Acute-on-Chronic Liver Failure

**DOI:** 10.1155/2021/5387856

**Published:** 2021-05-18

**Authors:** Xiaoyu Fu, Da Cheng, Yi Ouyang, Ying Li, Ronghua Li, Shifang Peng, Lei Fu

**Affiliations:** ^1^Department of Infectious Diseases, Hunan Key Laboratory of Viral Hepatitis, Xiangya Hospital, Central South University, Changsha, 410008 Hunan, China; ^2^Department of Nuclear Medicine, Xiangya Hospital, Central South University, Changsha, 410008 Hunan, China

## Abstract

People infected with chronic hepatitis B virus (HBV) might progress to acute-on-chronic liver failure (ACLF) with a high fatality rate. Long noncoding RNAs (lncRNAs) are involved in human diseases, but it is unknown whether lncRNAs are involved in the progression of chronic HBV infection to ACLF. Hence, this study is aimed at systemically identifying and characterizing the landscape and the molecular mechanism of lncRNAs in the pathogenesis of chronic HBV infection progress to ACLF. RNA sequencing (RNA-Seq) of peripheral blood samples from 5 ACLF and 5 HBV infection patients was performed. We detected 9733 lncRNAs, including 406 annotated lncRNAs and 9327 novel lncRNAs. A total of 407 lncRNAs were found to be significantly dysregulated in the patients with ACLF as compared with those in the chronic HBV infection patients. The flanking protein-coding genes of differentially expressed lncRNAs were enriched with pathways that might contribute to the pathogenesis of ACLF, such as the WNT signaling pathway. Furthermore, 9 selected differentially expressed lncRNAs validated by the qRT-PCR, showing that the expression patterns of these 9 lncRNAs were consistent with the RNA-Seq data. Four selected differentially expressed lncRNAs were also validated in another patient cohort comprising 80 patients with ACLF and 65 patients with chronic HBV infection. Aberrant lncRNAs might be used to develop novel diagnostic biomarkers or drug targets for ACLF.

## 1. Introduction

Hepatitis B virus (HBV) is a global epidemic disease. According to a report from the World Health Organization, approximately 2 billion people worldwide are infected with HBV, with 240 million of them presenting chronic HBV infections. Furthermore, approximately 650 000 people died from liver failure, cirrhosis, and hepatocellular carcinoma (HCC) induced by HBV infection [1]. In China, 93 million people are infected with chronic HBV every year, and approximately 30% patients present spontaneous hepatitis acute exacerbation, with some patients progressing to acute-on-chronic liver failure (ACLF) with a high fatality rate. ACLF can lead to severe clinical syndromes such as jaundice, blood coagulation dysfunction, hepatic encephalopathy, and ascites [2, 3]. In recent years, experts have considered ACLF as a new type of liver disease with short-term mortality rate (generally 28 days) and dysfunctional systematic inflammatory response. It is difficult to distinguish the initial clinical symptoms between ACLF and severe chronic hepatitis. Moreover, the short-term case fatality rate reaches up to >50% when the disease progresses to liver failure [4]. Although liver transplantation is the only effective treatment approach for liver failure, most patients cannot benefit from liver transplantation owing to source limitation of donor liver, economic pressure, and operation risks [2]. Therefore, it is important to prevent the occurrence of liver failure during the early clinical period.

In China, the general cause of ACLF is hepatitis virus, mainly HBV (approximately 80–85%). Patients with chronic HBV have a long progression window from acute exacerbation of hepatitis to ACLF, which is known as “acute on-chronic pre-liver failure” (pre-ACLF) [3]. The initial clinical manifestations are highly similar between chronic HBV infection and ACLF. With the dynamic development of the disease, some patients might recover gradually from severe chronic hepatitis, without liver failure, whereas others might develop to ACLF due to further aggravation of liver failure. The pathogenesis of ACLF is extremely complex and is affected by various factors, but the specific mechanism is not known. Thus, it is important to discover molecular mechanisms underlying the progression of chronic HBV infection to ACLF, identify clinical biomarkers to predict or diagnose in time, and assist in the development of necessary intervention and treatment for reducing the risk of death in patients with ACLF.

A large fraction of human transcripts is long noncoding RNAs (lncRNAs), as only approximately 1% of human transcripts encode proteins [5, 6]. The lncRNAs are spliced, polyadenylated transcripts, of size > 200 bp, and they lack the potential to encode proteins [7–9]. lncRNAs play important roles in several important biological processes, such as cell proliferation, differentiation, and apoptosis [6, 10–13]. A common example is the inactivation of X chromosome through cis-acting XIST lncRNA [14]. Recent studies have showed that dysfunctional lncRNAs might be involved in the development of liver diseases, such as liver fibrosis [15, 16]. For example, Song et al. have reported that the activation of hepatic lncRNA H19 could promote cholestatic liver fibrosis through the ZEB1-EpCAM signaling pathway in mice [15]. In addition, several lncRNAs have been shown to be dysregulated in HCC tissues, and they can act as potential biomarkers for diagnosing HCC and predicting the prognosis and response to therapy [17, 18]. For instance, lncRNA HULC is specifically expressed in hepatocytes and highly upregulated in liver cancer. It plays an important role in tumorigenesis and is associated with the intrahepatic metastases, HCC recurrence, and postoperative survival [19, 20]. Despite these findings of a few lncRNAs linked with liver diseases, it is not clear whether lncRNAs are involved in the pathogenesis of ACLF.

RNA sequencing (RNA-Seq) has been widely used to study whole transcriptome changes under different conditions, in order to quantify gene expression with dynamic range and overcome the shortcomings of the microarray technology [21, 22]. Recent advances in RNA-Seq and computational methods for reconstructing transcriptome offer an excellent opportunity to annotate and characterize lncRNAs, and a large number of lncRNAs have been identified using RNA-Seq. For example, Cabili et al. have identified more than 8 000 human long intergenic noncoding RNAs using RNA-Seq data and showed that most of them have not been previously described [13]. Therefore, abundant RNA-Seq data could enable to comprehensively identify and quantify lncRNAs (also protein-coding genes) under pathological conditions.

In this study, to explore the biological role of lncRNAs in the pathogenesis mechanism in the progression of chronic HBV infection to ACLF, we performed RNA-Sequencing of samples from 5 patients with ACLF and 5 patients with HBV infection (chronic asymptomatic HBV carrier, AsC). By analyzing the RNA-Seq data, we identified aberrantly expressed lncRNAs and protein-coding genes between ACLF and AsC patients. Moreover, the Gene Ontology (GO) and Kyoto Encyclopedia of Genes and Genomes (KEGG) pathway analyses were performed to predict the biological roles and potential signaling pathways of these differentially expressed lncRNAs. In addition, an lncRNA–mRNA network analysis was conducted to further explore the potential roles of differentially expressed lncRNAs in ACLF pathogenesis. Furthermore, we performed quantitative real-time PCR (qRT-PCR) analysis to confirm that the expression of 15 lncRNAs in another larger cohort comprising patients with ACLF due to HBV infection. The dysfunctional expression of lncRNAs might serve as potential biomarkers of ACLF, and aberrant lncRNAs involved in ACLF might open new avenues for the diagnosis and treatment of liver failure in human in the future.

## 2. Materials and Methods

### 2.1. Patients and Samples

From January 2016 to May 2017, 5 patients with ACLF and 5 patients with HBV (chronic asymptomatic HBV carrier, AsC) from the Xiangya Hospital of Centre-South University (Hunan, China) were enrolled in the present study. Blood samples were collected from all patients. These patients were all infected with HBV. Patients with liver damage caused by drinking, drugs, and other factors were excluded. 5 ACLF patients were diagnosed according to APSAL diagnostic criteria. Among them, 2 patients had not received nucleoside analogues (NAs) treatment, and HBV was in an active replication state in these 2 patients. 3 patients had received NA treatment, and the drugs were discontinued irregularly for more than half a year. All patients were treated with NAs after being diagnosed with ACLF. There were no patients with hepatic encephalopathy, and all these 5 ACLF patients were in the early stage of ACLF. The prognosis of 5 ACLF patients is improved and discharged. AsC exhibited HBsAg positive for more than 6 months, HBV DNA positive, and HBeAg negative, or positive. However, serum ALT and AST continue to be at normal levels. These patients were followed up 3 times in 1 year with an interval of more than 3 months. Patients with AsC were diagnosed and excluded as previous reported [23, 24]. The clinical characteristics of these 10 patients are summarized in Table 1 and Supplementary Table 2. We used these samples to perform RNA-Seq.

Another cohort, comprising 80 patients with ACLF and 65 patients with HBV infection were recruited from the Xiangya Hospital of Centre-South University (Hunan, China). These samples were used to verify the expression of differentially expressed lncRNAs through qRT-PCR. The clinical characteristics of these 145 patients are shown in Supplementary Table 3.

All individuals provided a written informed consent for use of their samples in this study. The present study was approved by the Ethics Committee of the Xiangya Hospital of Centre-South University (2017-P2-084-01).

### 2.2. RNA-Seq Library Preparation and Sequencing

The peripheral blood was obtained from the patients on the day of admission, and these patients had already suffered from ACLF at the time of admission. Next, the generation sequencing of RNA was performed using peripheral blood samples obtained from the patients. The total RNA was extracted from peripheral blood samples using Trizol reagent (Invitrogen, CA, USA), following the manufacturer's instruction. The RNA quantity and purity were analyzed using a Bioanalyzer 2100 and RNA 6000 Nano LabChip Kit (Agilent, CA, USA) with the RIN (RNA Integrity Number) > 7.0. Approximately 10 *μ*g of total RNA representing a specific adipose type was used to deplete the ribosomal RNA according to the instructions of the Epicentre Ribo-Zero Gold Kit (Illumina, San Diego, USA). Following purification, the poly (A) - or poly (A) + RNA fractions were fragmented into small pieces using divalent cations under elevated temperature. The cleaved RNA fragments were then reverse-transcribed to create the final cDNA library in accordance with the protocol of the mRNA-Seq Sample Preparation Kit (Illumina, San Diego, USA); the average insert size for the paired-end libraries was 300 bp (± 50 bp). We also performed paired-end sequencing on Illumina X10 (lc-bio, China), following the vendor's recommended protocol.

### 2.3. Human Gene Annotations

Human reference genome sequence of GRCh38 version was collected from the ENSEMBL database. Human gene annotations of protein-coding genes and lncRNAs of GRCh38 version were also downloaded from the ENSEMBL database.

### 2.4. Bioinformatics Analysis of the Transcriptome Data

For the raw RNA-Seq data, we firstly assessed the quality of sequencing reads using the Fastqc software (v 0.10.1) and then discarded low quality reads that showed quality score less than 10, contained the rates of base N more than 5%, or were contaminated by adaptors, as well as host sequences with 90% sequence similarity using Cutadpter (v 1.10) tool. Next, we aligned the cleaned reads of each sample to the GRCh38 reference genome through the TopHat software (v 2.0.4) [25], a gapped aligner capable of discovering splice junctions. The aligned reads from TopHat were then assembled into transcriptome, and the gene expression was quantified using StringTie (v 1.3.0) tool [26], which de novo assembles and quantitates full-length transcripts representing multiple splice variants for each gene locus.

### 2.5. Identification of Known and Prediction of Novel lncRNAs

For the identification of known and novel lncRNAs involved in ACLF, a bioinformatics computational pipeline was developed (Supplementary Figure 1). Firstly, all the transcripts assembled using StringTie were annotated with gene annotations of the GRCh38 protein-coding genes and lncRNAs, if the transcripts overlapped with the protein-coding genes, then the transcripts were considered as protein-coding genes. If the remaining transcripts overlapped with lncRNAs, then the transcripts were considered as known lncRNAs. The remaining unannotated transcripts were used for identifying potential novel lncRNAs. Secondly, these unannotated transcripts were filtered if the transcript length was less than 200 bp, the exon number was less than 2, and the coverage of reads number was less than 3. Finally, the remaining transcripts were evaluated for the coding potential using both Coding Potential Calculator (CPC) [27] and Coding-Non-Coding Index (CNCI) [28]. The transcripts with a CPC score < −1 and CNCI score < 0 were considered as potential novel lncRNAs.

### 2.6. Identification of Differentially Expressed Protein-Coding Genes and lncRNAs between the ACLF and AsC Groups

Ballgown algorithm was used to compare the ACLF and AsC groups to identify differentially expressed protein-coding genes and lncRNAs. The protein-coding genes and lncRNAs with ≥ 2-fold change in expression and statistical significance (*p* < 0.05) were considered as differentially expressed.

### 2.7. Functional Enrichment Analysis

The significance of enriched GO terms and KEGG pathways for the protein-coding genes was determined using the GOseq software (v1.18.0) and KOBAS (v2.0) [29]. The false discovery rate (FDR; corrected *p* value) of < 0.05 was set as the cutoff for significant GO terms and KEGG pathways.

### 2.8. The qRT-PCR Validation of lncRNAs

We selected top 15 differentially expressed lncRNAs to perform qRT-PCR to validate their expression in the 10 patients. In addition, nine lncRNAs were validated in another cohort, comprising 80 patients with ACLF and 65 patients with HBV infection. The total RNA was reversed transcribed into cDNA using SuperScript III Reverse Transcriptase (Invitrogen, Grand Island, NY, USA) in accordance with the manufacturer's instructions. An Applied Biosystems ViiA 7 Real-Time PCR System and 2 × PCR Master Mix were used to perform the qRT-PCR (Arraystar). The reaction conditions were as follows: incubation at 95°C for 10 min, followed by 40 cycles of 95°C for 10 s and 60°C for 1 min. The relative lncRNA expression was calculated using the 2^-*ΔΔ*Ct^ method and was normalized to that of GAPDH, as an endogenous reference transcript [30]. The specific primers for each lncRNAs are listed in Table 2.

### 2.9. Target Gene Prediction and Functional Analysis of lncRNAs

lncRNAs might play a cis-acting element role on the neighboring target genes. To explore the function of lncRNAs, we predicted the cis-target genes of lncRNAs. In this study, the coding genes 100000 bp upstream and downstream were collected. The functional analysis of the target genes for lncRNAs was then performed using BLAST2GO [31]. A function was considered significantly enriched if the *p* value was < 0.05 by using a hypergeometric test.

## 3. Results

### 3.1. Expression Landscape of lncRNAs during the Progression of AsC to ACLF

ACLF is a serious threat to human health. Therefore, we aimed to explore the pathogenesis mechanism of ACLF and characterize a comprehensive expression landscape of lncRNAs during the progression of AsC to ACLF. By aligning the sequenced reads to human reference sequences, we found that on an average 95 million unique reads per samples were mapped to human reference sequence (Supplementary Table 1). To depict the landscape of lncRNAs during the progression of AsC to ACLF, we performed a bioinformatics analysis using the transcriptome data. First, we used StringTie tool to assemble the aligned reads to transcripts. The assembled transcripts were then annotated with annotations of the GRCh38 protein-coding genes and lncRNAs to separate the protein-coding genes and known lncRNAs. If the transcripts were not overlapped with the protein-coding genes but overlapped with the annotated lncRNAs, then the transcripts were considered as known lncRNAs. To characterize a comprehensive profile of lncRNAs, the remaining unannotated transcripts were used for identifying potential novel lncRNAs (Supplementary Figure 1). Briefly, the unannotated transcripts were discarded by length (< 200 bp), the exon number (< 2), and the coverage of reads number (< 3). Finally, the remaining transcripts were predicted for the coding potential using both CPC and CNCI algorithm. The transcripts with a CPC score < −1 and CNCI score < 0 were considered as potential novel lncRNAs.

We identified 9733 lncRNAs during the progression of AsC to ACLF, including 406 known lncRNAs and 9327 novel lncRNAs. Moreover, some well-known lncRNAs were expressed during the progression of AsC to ACLF, such as lncRNA GAS5, MALAT1, and NEAT1. To depict the features of lncRNAs, we compared the lncRNAs and protein-coding genes (Supplementary Figure 2). The number of expressed lncRNAs was less than that of the protein-coding genes (Supplementary Figure 2A). The expression level of lncRNAs was comparable with that of the protein-coding genes. In addition, the exon number and length of the protein-coding genes were more than those of the lncRNAs (Supplementary Figure 2B, C). However, the predicted open reading frame (ORF) length of lncRNAs was less than that of the protein-coding genes (Supplementary Figure 2D, lncRNAs mean ORF: 83; protein-coding genes mean ORF: 316), which is consistent with the findings of previous studies that lncRNAs have relatively lower coding potential [10, 13]. Overall, these results suggested that lncRNAs play an important role in the transcriptional landscape of the hepatic gene expression and the dysfunctional expression of lncRNAs might be involved in the progression of AsC to ACLF.

### 3.2. Differentially Expressed lncRNAs in the Progression of AsC to ACLF

**Figure 1:**
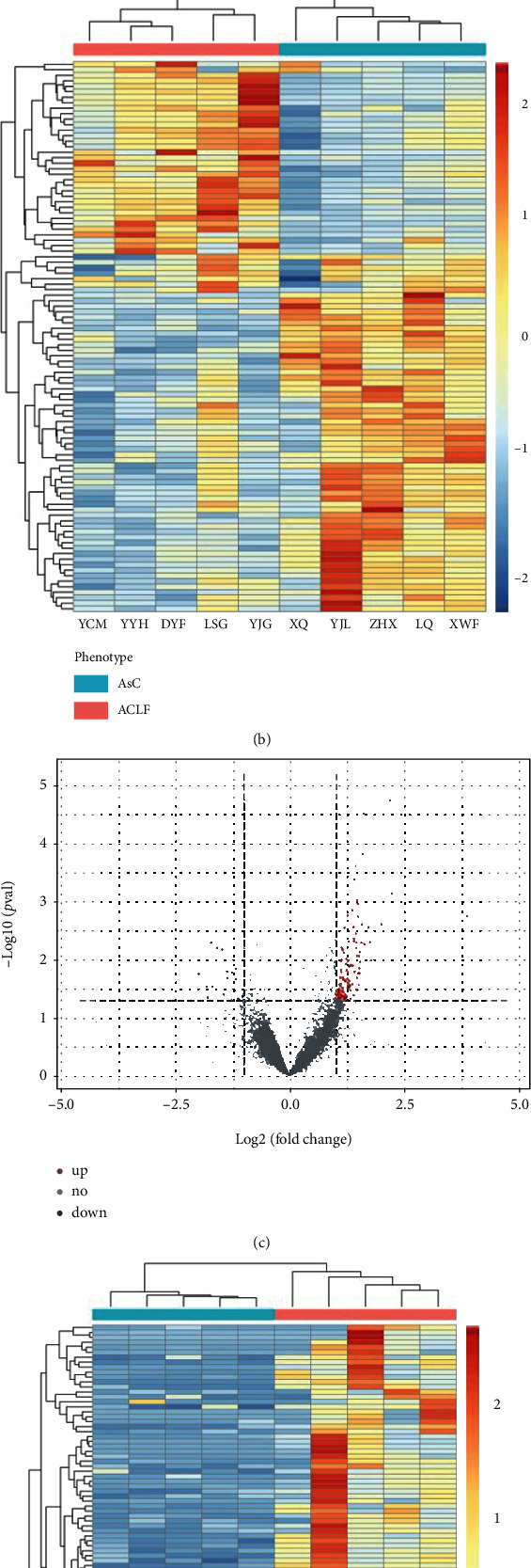
Differentially expressed lncRNAs in the progression of AsC to ACLF. (a) A volcano plot illustrating the fold changes and *p* values for protein-coding genes. (b) A heat map of top 100 differentially expressed protein-coding genes. (c) A volcano plot illustrating the fold changes and *p* values of lncRNAs. (d) A heat map of top 100 differentially expressed lncRNAs.

To identify lncRNAs differentially expressed in the ACLF group as compared with that in the AsC group, we performed a differential expression analysis using Ballgown algorithm, which is considered a robust method to analyze the RNA-Seq data [32]. We found that 768 differentially expressed protein-coding genes with ≥2 fold change between the ACLF and the AsC groups (FDR < 0.001), including 483 upregulated and 285 downregulated protein-coding genes (Figures 1(a) and 1(b)). For lncRNAs, we obtained 407 differentially expressed lncRNAs in the ACLF group by comparing with those of the AsC group, including 155 upregulated and 252 downregulated lncRNAs (Figure 1(c)). Based on the differentially expressed lncRNAs, we could clearly divide the ACLF and AsC groups (Figure 1(d)). For example, the lncRNA THRB showed a 3.2-fold upregulation in the ACLF group compared with that in the AsC group. In summary, the aberrant expression of lncRNAs might be involved in the pathogenesis of the progression of AsC to ACLF.

### 3.3. Validation of Differentially Expressed lncRNAs Using qRT-PCR

**Figure 2:**
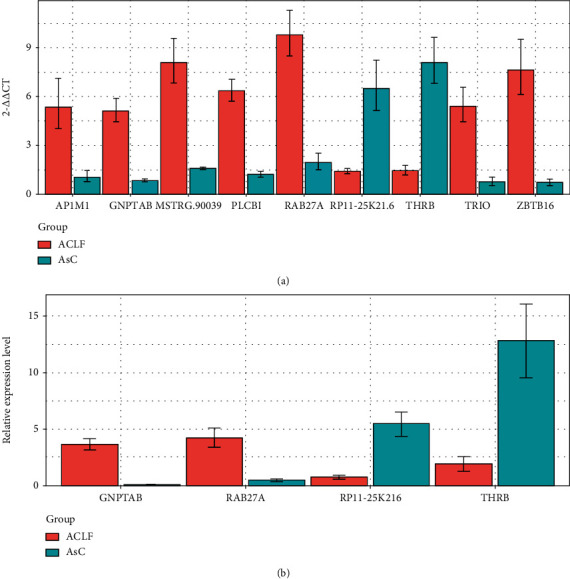
Validation of differentially expressed lncRNAs by qRT-PCR with statistical significance in the cohort comprising 5 patients with ACLF and 5 patients with hepatitis B virus (a) and another larger cohort comprising 80 patients with ACLF and 65 patients with AsC (b).

To demonstrate the expression of lncRNAs, we selected the top 15 differentially expressed lncRNAs (Table 2) with a variable fold change to validate by qRT-PCR. The results revealed that the expression of nine lncRNAs was consistent with the RNA-Seq data (Figure 2(a), Supplementary Figure 3A). Furthermore, we compiled a larger patient cohort comprising 80 patients with ACLF and 65 patients with HBV to perform the qRT-PCR experiment to validate the expression of the nine selected differentially expressed lncRNAs. The results were consistent with the change in gene expression observed in the previous cohort. As shown in Figure 2(b), we also found that four lncRNAs (RP11-25K21.6, THRB, RAB27A, and GNPTAB) out of nine lncRNAs (Supplementary Figure 3B) were significantly differentially expressed between the ACLF and the AsC groups. The lncRNAs RP11-25K21.6 and THRB were downregulated in the ACLF group compared with those in the AsC group. Whereas, the expression of lncRNAs RAB27A and GNPTAB was enhanced in the ACLF group with respect to the AsC group. In conclusion, we confirmed the abnormal expression of lncRNAs using RNA-Seq data by qRT-PCR experiments in a larger patient cohort. The results suggested that the aberrant expression changes in lncRNAs could play an important role in the progression of AsC to ACLF and that these lncRNAs might serve as potential biomarkers of ACLF, which has to be further studied.

### 3.4. Target Gene Prediction and Functional Analysis of Differentially Expressed lncRNAs

**Figure 3:**
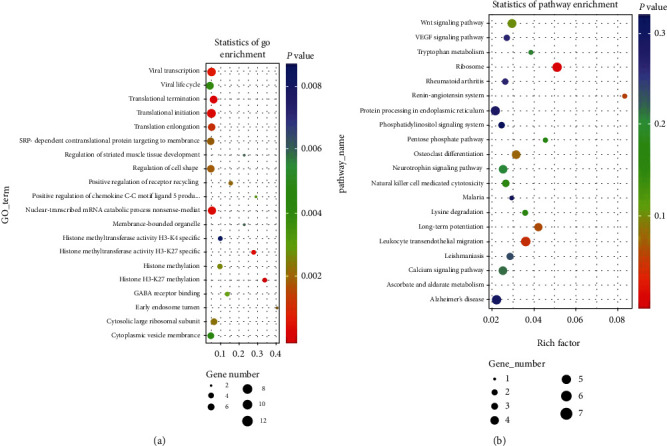
Significantly enriched GO terms (a) and KEGG pathways (b) of differentially expressed lncRNAs. GO term and KEGG pathway enrichment analyses of differentially expressed lncRNAs were performed using adjacent protein-coding genes located within the flanking 100 kb.

The regulation of lncRNAs can be classified into two groups: the first is cis-regulation, wherein lncRNA can regulate neighboring protein-coding genes, and the second is trans-regulation. The biological functions of differentially expressed lncRNAs in ACLF patients are not known; therefore, we predicted the targets of differentially expressed lncRNAs using previously described methods [13]. We defined the potential protein-coding gene targets as those located within the 100 kb flanking regions of differentially expressed lncRNAs. Then, based on these adjacent protein-coding genes, we predicted the functions of the differentially expressed lncRNAs. As shown in Figure 3, GO term and KEGG pathway enrichment indicated that the adjacent protein-coding genes were enriched for functions such as transcription and translation initiation. GO analysis revealed that the histone methylation function was significantly enriched. Zhang et al. have found that suppressor of cytokine signaling 1 gene promoter methylation is associated with acute-on-chronic hepatitis B liver failure [33]. Thus, the differentially expressed lncRNAs flanking these methylation associated protein-coding genes may also play an important role in mediating liver failure of ACLF patients. In addition, adjacent protein-coding genes were significantly enriched for the WNT signaling pathway. It is known that the WNT signaling pathway plays crucial roles in various biological processes, such as development, proliferation, differentiation, growth, and regeneration [34, 35]. Accumulating evidences have shown that the activated WNT signaling pathway is involved in liver diseases, such as liver fibrosis, and in liver cell regeneration [34, 36]. Our findings implied that lncRNAs may regulate adjacent protein-coding WNT genes, dysregulate the WNT signaling pathway, involved in the pathogenesis of ACLF. In total, based on the adjacent protein-coding genes of differentially expressed lncRNAs, we found that these lncRNAs may be involved in key biological functions and pathways which participate in the progression of AsC to ACLF.

**Figure 4:**
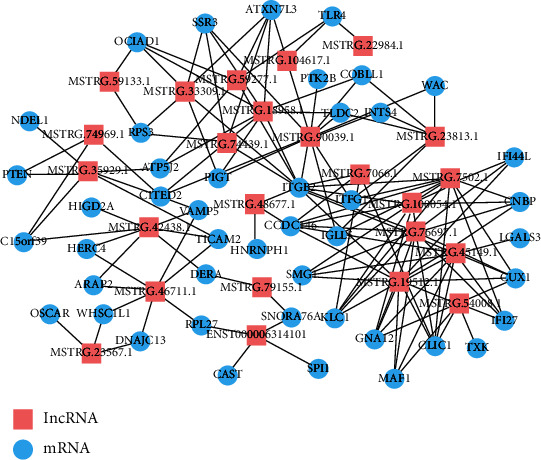
The network of lncRNAs and protein-coding genes.

A potential cis network was constructed between differentially expressed lncRNAs and protein-coding genes based on the predicted target relationships between them. As shown in Figure 4, the whole network consisted of 69 network nodes and 150 connections among 45 protein-coding genes and 24 lncRNAs. Moreover, our data showed that one mRNA might be regulated by multiple lncRNAs, and at the same time, one lncRNA may also regulate multiple protein-coding genes, suggesting complex regulatory relationships between lncRNAs and protein-coding genes.

## 4. Discussion

Chronic HBV infection is tightly associated with the occurrence of ACLF. However, the underlying pathogenesis of the progression from chronic HBV infection to ACLF is not fully understood. Accumulating evidences have shown that lncRNAs play key roles in the pathways underlying liver diseases or liver cancer [16, 19, 37–39]. For example, the activation of hepatic lncRNA H19 promotes cholestatic liver fibrosis through ZEB1-EpCAM signaling pathway in mice [15]. lncRNA HULC is specifically and highly upregulated in liver cancer, and it is associated with intrahepatic metastases, HCC recurrence, and postoperative survival [19, 20]. We wondered whether lncRNAs can be involved in the progression from chronic HBV infection to ACLF, and which lncRNA can be used as potential biomarkers to predict ACLF. Therefore, analyzing the expression profiles of lncRNAs could provide new insights for understanding the aetiology and pathophysiology of the progression from chronic HBV infection to ACLF.

In this study, we used a deep sequencing technology to characterize the expression landscape of lncRNAs in 5 ACLF and 5 chronic HBV infection patients. We analyzed differential expression of lncRNAs and predicted target protein-coding genes to identify ACLF-associated differentially expressed lncRNAs and to infer the potential functional relevance of these lncRNAs during the progression from chronic HBV infection to ACLF.

Through transcriptome analysis, we obtained 9733 lncRNAs including 406 known lncRNAs and 9327 novel lncRNAs during the progression of AsC to ACLF. Some well-known lncRNAs, such as lncRNA GAS5, MALAT1, and NEAT1, were expressed. In agreement with the results of previous studies [40, 41], we observed higher variability in the number of expressed lncRNAs than in protein-coding genes. However, the exon number, transcript length, and expression levels of lncRNAs were comparable with that of protein-coding genes. The predicted ORF length of lncRNAs was also less than that of protein-coding genes, and this result was consistent with the lower coding potential of lncRNAs described in the previous research [13].

Through comparison of ACLF patients and hepatitis B virus carriers, we found 407 lncRNAs differentially expressed in ACLF, including 155 upregulated and 252 downregulated lncRNAs. On the basis of the differentially expressed lncRNAs, the ACLF patients and hepatitis B virus carriers could be divided into two distinct groups. For example, lncRNA THRB showed 3.2-fold higher expression in ACLF patients than in hepatitis B virus carriers. Then, we chose 15 differentially expressed lncRNAs and performed qRT-PCR experiments to further validate the expression of these lncRNAs in the two patient cohorts. Nine out of the 15 differentially expressed lncRNAs showed similar expression levels between qRT-PCR experiments and in RNA-Seq data. Four out of these 9 differentially expressed lncRNAs also exhibited significant differential expression between 80 ACLF patients and 65 hepatitis B virus carriers by qRT-PCR. lncRNA RP11-25K21.6 and THRB were downregulated, and lncRNA RAB27A and GNPTAB were upregulated in ACLF patients compared with hepatitis B virus carriers. Thus, the aberrant expression of lncRNAs may be involved in the progression of AsC to ACLF. These findings could lead towards a better understanding of the function of dysregulated lncRNAs in the etiology of the progression from chronic HBV infection to ACLF.

Functional enrichment analysis revealed that the significantly differentially expressed lncRNAs were involved in histone methylation, the WNT signaling pathway, and other processes. In a previous study, methylation of the suppressor of cytokine signaling 1 gene promoter has been demonstrated to be associated with acute-on-chronic hepatitis B liver failure [33]. Thus, differentially expressed lncRNAs flanking these methylation-associated protein-coding genes may also play an important role in liver failure of ACLF patients. The WNT signaling pathway is involved in liver diseases and liver cell regeneration, such as liver fibrosis [34]. In our study, we found that the differentially expressed lncRNAs flanking protein-coding genes were involved in the WNT signaling pathway, implying that lncRNA may regulate adjacent protein-coding genes to induce the aberrant expression of WNT signaling pathway components, which is involved in the pathogenesis of ACLF. These findings have suggested that the differentially expressed lncRNAs may play a role in key pathways involved in the progression of AsC to ACLF. We also constructed a network of differentially expressed lncRNA and protein-coding gene consisting of 45 protein-coding genes and 24 lncRNAs and found that protein-coding genes may be regulated by multiple lncRNAs, and one lncRNA may also regulate multiple protein-coding genes.

Accumulation studies have reported that many lncRNAs participate in the HBV propagation. For example, lncRNA HOTAIR can regulate transcription and replication of HBV through promoting SP1 transcription factor [42]. lncRNA PCNAP1 promotes HBV replication and accelerates hepatocarcinogenesis via miR-154/PCNA/HBV covalently closed circular DNA signaling pathways [43]. In HBV-related hepatocellular carcinoma, lncRNA activates HBV through regulating HBx/STAT3/miR-539/APOBEC3B axis, thereby promoting the progression of HBV-related hepatocellular carcinoma [44]. Thus, lncRNA is essential for HBV propagation. Additionally, Jiang et al. have confirmed that 132 novel lincRNAs whose expression levels are globally associated with ages [45]. Due to the small sample size, the ages of patients in our study were quite different between the AsC group and the ACLF group. Thus, the abnormal expression of lncRNAs may be related to ages of patients. This is a shortcoming in our research. The underlying connection among lncRNA, age, and ACLF still needs further research.

## 5. Conclusions

In conclusion, our study provides a novel compendium of lncRNAs expressed in the progression of chronic HBV infection to ACLF. We characterized the expression landscape of lncRNAs and identified the expression patterns of lncRNAs significantly altered in ACLF. Compared with the patients with chronic HBV infection, there were 407 lncRNAs were significantly dysregulated in ACLF patients, including 155 upregulated and 252 downregulated lncRNAs. These abnormal expressed lncRNAs may participate in the progression of ACLF. Furthermore, we validated these altered expression patterns by qRT-PCR in two patient cohorts, including RP11-25K21.6, THRB, PLCB1, TRIO, DOK6, APTX, ST3GAL4, MSTRG.7502, ZBTB16, MSTRG.90039, PAPSS1, ERN1, AP1M1, RAB27A, and GNPTAB. Aberrantly expressed lncRNAs were found to be involved in specific biological processes and related pathways that could contribute to the pathogenesis of ACLF. The results of the present study may provide a foundation for future study of the role of lncRNAs in the progression of chronic HBV infection to ACLF. Identification of dysregulated lncRNAs involved in this progression may contribute to the development of novel diagnostic biomarkers or drug targets for the treatment of ACLF. However, further research is required to determine the detailed molecular mechanisms underlying the action of significantly dysregulated lncRNAs in ACLF.

## Figures and Tables

**Table 1:** Clinical characteristics of 10 patients.

Groups	Gender	Age	ALB (g/L)	TBIL (*μ*mol/L)	DBIL (*μ*mol/L)	TBA (*μ*mol/L)	ALT (U/L)	AST (U/L)
AsC group	F	28	48.2	11.6	6.4	8.2	33.5	19
	F	31	50.3	12.2	5.8	10.1	29.7	20.8
	M	27	53.8	9.1	3.9	1.7	32.8	23.6
	M	33	47.7	10.5	4.9	5	20.5	21.2
	M	39	46.9	9.8	5.3	3.9	19.7	17.3
ACLF group	M	36	21.7	307.3	178.5	101.4	409.3	189.7
	M	52	35.8	618.1	321.7	137.3	322.5	237.3
	F	50	34	508.8	260.4	189.8	707.1	433.4
	F	45	31.1	376.4	147.5	126.9	609.4	176.1
	M	63	29.6	679.6	319.3	132.3	1033.5	770.2

**Table 2:** lncRNA primer sequences for qRT-PCR.

Number	lncRNA	Sequence (5′ to 3′)
1	lncRNA RP11-25K21.6-F	GCGTTGGTGGTATAGTGGTGAGC
	lncRNA RP11-25K21.6-R	ACACAGCGCGTTATAGGTTCTGAC
2	lncRNA THRB-F	GCACTTGAGACACTCTGGTCGTTC
	lncRNA THRB-R	GCCACATCTCATCCAGACCACTTG
3	lncRNA PLCB1-F	ACCAGTGGGATGGTAGAAGGT
	lncRNA PLCB1-R	TCTTTGCTAGCTGTTTAGCACG
4	lncRNA TRIO-F	CTGCCAGTTGTTCTTTTGCAGG
	lncRNA TRIO-R	GGGAAAGTAAGGGACTCGGGA
5	lncRNA DOK6-F	TGTAGCCTGAGCATCCCCTT
	lncRNA DOK6-R	ATCCTGGAGGGCTTGGATGG
6	lncRNA APTX-F	GTTTATGGGACAGGTGATACCTCA
	lncRNA APTX-R	ACAGGGTTGCTAAGATGATGAAATG
7	lncRNA ST3GAL4-F	CAGATCCCAGCTCAAAGGCG
	lncRNA ST3GAL4-R	TTCGGGAGAGCACTCAGAGG
8	lncRNA MSTRG.7502-F	CTTGCTCATCGATTCCAATGATTCC
	lncRNA MSTRG.7502-R	CTTTCCAAGTTTTGCCCCCTG
9	lncRNA ZBTB16-F	GGAGCTCAACAGGCACACAA
	lncRNA ZBTB16-R	AGCAGCAGCATTGTGACTCC
10	lncRNA MSTRG.90039-F	TCATGGGGTCGGTACCAAGG
	lncRNA MSTRG.90039-R	CATCTGCACTGGAGGAGGGA
11	lncRNA PAPSS1-F	TCCTCCTTTGTGGCAGACCA
	lncRNA PAPSS1-R	TGCTCACTCACCCGACCCTT
12	lncRNA ERN1-F	TTGTCCATTCCTGCCTGGGA
	lncRNA ERN1-R	CAAGTGGTCGGCAGGAAACA
13	lncRNA AP1M1-F	TACCACGCCTGGCCTATTCC
	lncRNA AP1M1-R	AGAGGACTTCTATCTGGAACACACA
14	lncRNA RAB27A-F	GGTGAGCATTTGGACTGGTTCC
	lncRNA RAB27A-R	AGTGGTTCCAATTTCCCCTCCT
15	lncRNA GNPTAB-F	GAAGGGGCAAGAATGGCTGC
	lncRNA GNPTAB-R	TCTCTTCCTATGTCCTTCACATGAC
16	GAPDH-F	AGAAGGCTGGGGCTCATTTG
	GAPDH-R	AGGGGCCATCCACAGTCTTC

## Data Availability

The data used to support the findings of this study are available from the corresponding author upon request.

## Supplementary Materials

Supplementary 1 Supplementary Figure 1: a bioinformatics computational pipeline was developed for identification of known and novel lncRNAs involved in ACLF.

Supplementary 2 Supplementary Figure 2: comparison of lncRNAs and mRNAs. (A) Expression levels of lncRNAs and mRNAs; (B) Transcript length of lncRNAs and mRNAs; (C) Exon numbers of lncRNAs and mRNAs; (D) ORFs length of lncRNAs and mRNAs.

Supplementary 3 Supplementary Figure 3: validation of differentially expressed lncRNAs by qRT-PCR without statistical significance in the patient cohort of 5 ACLF and 5 hepatitis B virus carrier (A) and a new patient cohort including 80 ACLF and 65 AsC patients (B).

Supplementary 4 Supplementary Table 1: the RNA-Seq data and mapping rates for the 10 samples.

Supplementary 5 Supplementary Table 2: clinical characteristics of 10 patients.

Supplementary 6 Supplementary Table 3: clinical characteristics of 145 patients.
