# Supplementary figures and images for: Integrated Analysis of Long Noncoding RNA Expression Profiles in Acute-on-Chronic Liver Failure
Source: Biomed Res Int. 2021 May 18;2021:5387856. doi: 10.1155/2021/5387856 (PMC8158414; doi:10.1155/2021/5387856)

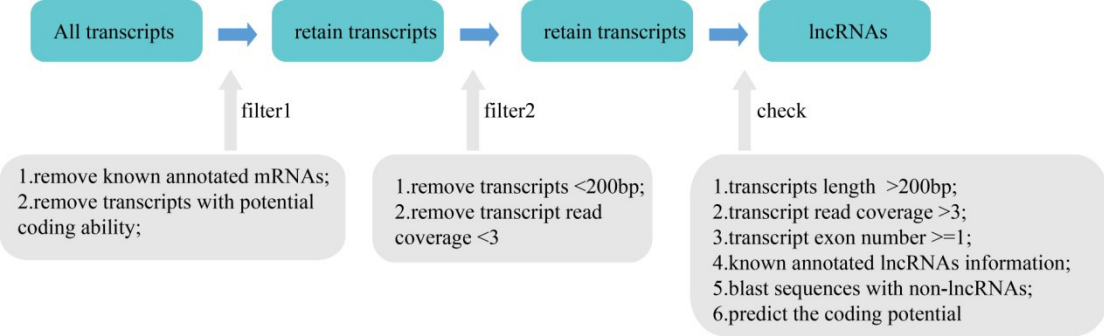

Supplement: Supplementary 1 — Supplementary Figure 1: a bioinformatics computational pipeline was developed for identification of known and novel lncRNAs involved in ACLF. [file 5387856.f1.pdf]

**A**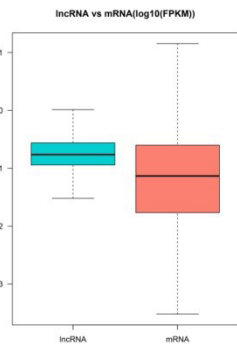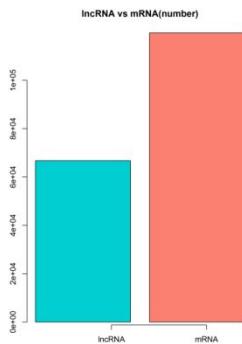**B**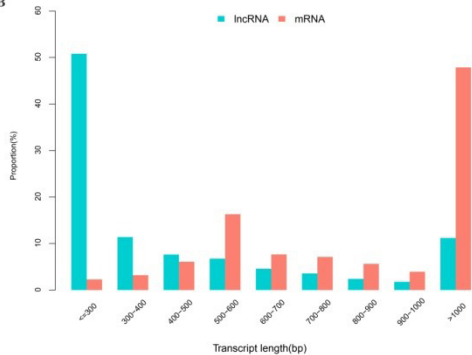**C**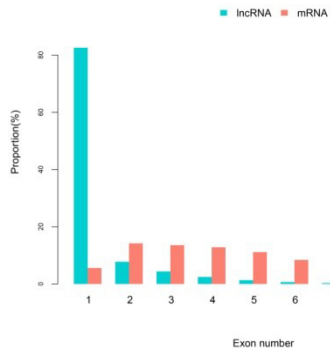**D**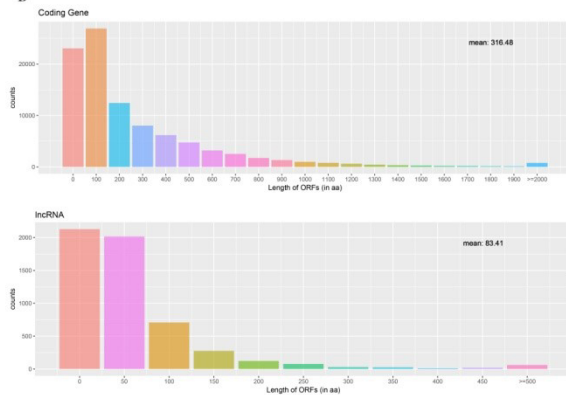

Supplement: Supplementary 2 — Supplementary Figure 2: comparison of lncRNAs and mRNAs. (A) Expression levels of lncRNAs and mRNAs; (B) Transcript length of lncRNAs and mRNAs; (C) Exon numbers of lncRNAs and mRNAs; (D) ORFs length of lncRNAs and mRNAs. [file 5387856.f2.pdf]

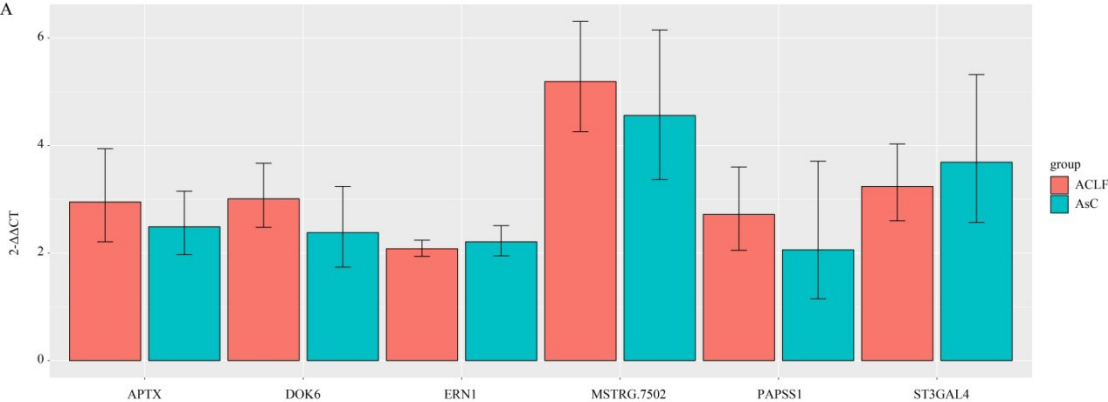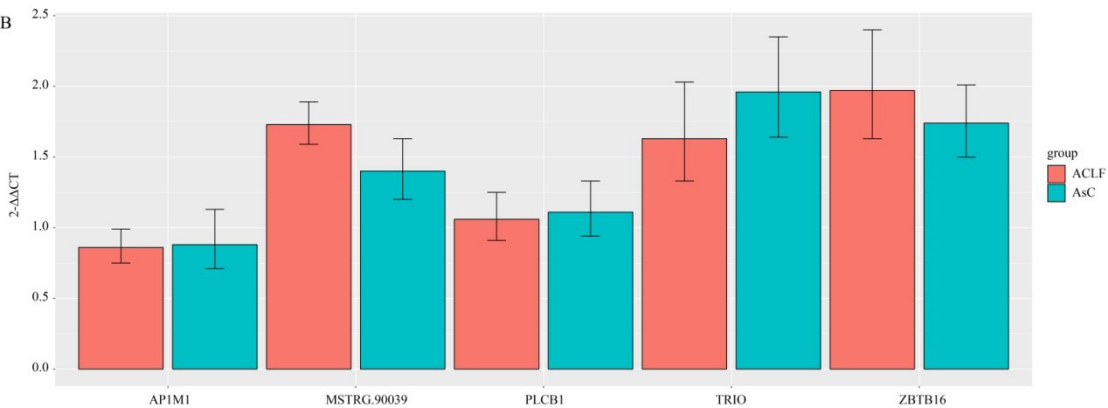

Supplement: Supplementary 3 — Supplementary Figure 3: validation of differentially expressed lncRNAs by qRT-PCR without statistical significance in the patient cohort of 5 ACLF and 5 hepatitis B virus carrier (A) and a new patient cohort including 80 ACLF and 65 AsC patients (B). [file 5387856.f3.pdf]
