# Supplementary material for: Integrated Analysis of Long Noncoding RNA Expression Profiles in Acute-on-Chronic Liver Failure
Source: Biomed Res Int. 2021 May 18;2021:5387856. doi: 10.1155/2021/5387856 (PMC8158414; doi:10.1155/2021/5387856)
Supplement: Supplementary 4 — Supplementary Table 1: the RNA-Seq data and mapping rates for the 10 samples. [file 5387856.f4.docx]

**Supplementary Table 1.** The RNA-seq data and mapping rates for the 10 samples.

| Sample | Group | Raw Data | Valid reads | Mapped reads | Unique Mapped reads |
| --- | --- | --- | --- | --- | --- |
| YJL | AsC | 96219248 | 86371242 | 58073944(67.24%) | 45626474(52.83%) |
| ZHX | AsC | 92860528 | 81088410 | 61772544(76.18%) | 48746661(60.12%) |
| LQ | AsC | 91868778 | 79876422 | 62353689(78.06%) | 53520767(67.00%) |
| XWF | AsC | 101385544 | 90110212 | 71763914(79.64%) | 63842349(70.85%) |
| XQ | AsC | 92628760 | 77947230 | 61779077(79.26%) | 49244121(63.18%) |
| LSG | ACLF | 85775144 | 72959746 | 57658002(79.03%) | 46315323(63.48%) |
| YYH | ACLF | 99573456 | 84550056 | 67139713(79.41%) | 54921537(64.96%) |
| YJG | ACLF | 89569396 | 55413564 | 44043506(79.48%) | 37128196(67.00%) |
| DYF | ACLF | 86932914 | 67959388 | 54546993(80.26%) | 45066707(66.31%) |
| YCM | ACLF | 85618680 | 73422798 | 60149521(81.92%) | 49645045(67.62%) |
