# Supplementary material for: Integrated Analysis of Long Noncoding RNA Expression Profiles in Acute-on-Chronic Liver Failure
Source: Biomed Res Int. 2021 May 18;2021:5387856. doi: 10.1155/2021/5387856 (PMC8158414; doi:10.1155/2021/5387856)
Supplement: Supplementary 5 — Supplementary Table 2: clinical characteristics of 10 patients. [file 5387856.f5.docx]

**Supplementary Table 2. Clinical characteristics of 10 patients**

| Groups | Gender | PT | INR | Cr (umol/L) |
| --- | --- | --- | --- | --- |
| AsC group | F | 10.01 | 0.88 | 88.3 |
|  | F | 11.6 | 1.04 | 76.4 |
|  | M | 12.33 | 1.09 | 101.2 |
|  | M | 13.2 | 1.14 | 64.8 |
|  | M | 11.06 | 0.98 | 70.5 |
| ACLF group | M | 17.43 | 1.54 | 62 |
|  | M | 18.47 | 1.67 | 59.4 |
|  | F | 19.24 | 1.71 | 66.2 |
|  | F | 19.46 | 1.75 | 70.4 |
|  | M | 19.93 | 1.77 | 50.9 |
