# Supplementary material for: Integrated Analysis of Long Noncoding RNA Expression Profiles in Acute-on-Chronic Liver Failure
Source: Biomed Res Int. 2021 May 18;2021:5387856. doi: 10.1155/2021/5387856 (PMC8158414; doi:10.1155/2021/5387856)
Supplement: Supplementary 6 — Supplementary Table 3: clinical characteristics of 145 patients. [file 5387856.f6.docx]

**Supplementary Table 3. Clinical characteristics of 145 patients**

| Groups | Female (n) | Male (n) | Age (year) | ALB (g/L) | TBIL (µmol/L) | DBIL (µmol/L) | TBA (µmol/L) | ALT (U/L) | AST (U/L) |
| --- | --- | --- | --- | --- | --- | --- | --- | --- | --- |
| ACLF group | 26 | 54 | 49 ± 18 | 36 ± 9.2 | 409.9 ± 213.4 | 257.65 ± 153.65 | 386.2 ± 240.5 | 442.2 ± 389.9 | 437.4 ± 395.5 |
| AsC group | 17 | 48 | 38.5 ± 17.5 | 43.7 ± 6.4 | 11.5 ± 6.5 | 6.6 ± 3.6 | 7.85 ± 4.35 | 24.95 ± 14.15 | 25.35 ± 19.15 |
